# A Review of Reviews of Patient-Reported Measures in Psychosis: Need to Consider Factors Affecting Equity and the Involvement of Patients

**DOI:** 10.1093/schizbullopen/sgae032

**Published:** 2025-01-11

**Authors:** Neha Nair, Maria Abou Farhat, Navdeep Kaur, Nev Jones, Greeshma Mohan, Jill Boruff, Srividya N Iyer

**Affiliations:** Department of Psychiatry, McGill University, Montreal, H3A 1A1, Canada; Douglas Mental Health University Institute, Montreal, H4H 1R3, Canada; Douglas Mental Health University Institute, Montreal, H4H 1R3, Canada; Douglas Mental Health University Institute, Montreal, H4H 1R3, Canada; School of Social Work, University of Pittsburgh, Pittsburgh, 15260, United States; Schizophrenia Research Foundation, Chennai, 600102, India; Schulich Library of Physical Sciences, Life Sciences, and Engineering, McGill University, Montreal, H3A 1G3, Canada; Department of Psychiatry, McGill University, Montreal, H3A 1A1, Canada; Douglas Mental Health University Institute, Montreal, H4H 1R3, Canada

**Keywords:** health equity, patient-reported measures, psychosis, schizophrenia, social determinants, lived experience

## Abstract

**Background:**

Patient-reported measures are increasingly valued in psychosis care and research. For patient-reported measures to reflect patient perspectives, patients must be involved in developing them. Furthermore, their development and evaluation must consider sociodemographic characteristics influencing patient experiences and outcomes and measurement. As reviews reflect the state of the field and guide clinicians/researchers in selecting measures, our aim was to evaluate literature reviews of patient-reported measures on their consideration of factors affecting equity and patient involvement.

**Study Design:**

For this review of reviews, we searched 3 databases (MEDLINE, Embase, and PsycINFO) for reviews on patient-reported measures in psychosis. Two reviewers independently screened titles, abstracts, and full texts, and descriptively synthesized and appraised the quality of included reviews. Using Cochrane’s PROGRESS-Plus and a Canadian equity framework, reviews were evaluated on their consideration of sociodemographic characteristics, accessibility, and patient involvement.

**Study Results:**

Of 10 reviews (6 systematic, 4 nonsystematic; 1111 studies; 313 measures), 6 limited their search to English. Barring 2 reviews that reported the age, gender, and countries of samples in included studies, the reviews did not extract/comment on population/sociodemographic characteristics. One commented on one measure’s readability; none commented on the samples’ literacy levels. Four reviews considered the availability of translations; only 1 evaluated cross-cultural validity. Only 2 considered the costs of measures. Only 1 evaluated patient involvement in developing patient-reported measures. One referenced equity frameworks/standards.

**Conclusions:**

Reviews of patient-reported measures in psychosis demonstrate minimal attention to equity and patient involvement. We offer recommendations to strengthen patient-reported measures research by attending to equity, social determinants, and patient-centrism.

## Introduction

Patient-reported outcome measures (PROMs) and patient-reported experience measures (PREMs) provide a rich source of information about health status, outcomes, and experience from the patient’s perspective.^1^ Since they can give patients and families a voice in their care and provide different and more ecologically valid information than that gathered from the clinician’s perspective, patient-reported measures can improve alliances, treatment, decision-making, and care quality.^2,3^

Historically, individuals diagnosed with psychosis or schizophrenia were thought to lack “insight” into their condition and consequently, were seen as incapable of usefully completing self-reports.^4,5^ More recently, however, it has been noted that poor “insight” (a concept which itself has been problematized) need not affect the validity of self-report measures in populations with psychosis.^6,7^ Furthermore, the rise of the recovery movement and patient-oriented/patient-centered research has contributed to an epistemological shift toward valuing patient-reported measures and increasing their use in clinical trials and measurement-based care systems in mental health.^8,9^

Particularly if co-developed with service users, patient-reported measures have the potential to make patient values, priorities, preferences, and perspectives visible. However, if their development, examination, and use neglect to consider and incorporate patient perspectives and values, PROMs and PREMs may *de facto* differ little from traditional instruments and still reflect clinician, rather than patient, priorities.^3,10^

When considering PROMs for use in different populations, it is important not to assume that a measure will automatically tap into similar constructs across cultures, languages, and geographical regions. Self-reported measures may be more or less valid and valuable depending on their readability and respondents’ ability to understand and accurately complete them^11^; and, particularly in translated versions, their sensitivity and cultural relevance.^12^

Because the populations availing healthcare are highly diverse across, as well as increasingly within, geographies, it is important that PROMs and PREMs be developed by, tested in, and validated for demographically, linguistically, geographically, and socioeconomically diverse communities and stakeholders. Doing so would allow for more accurate assessment and appropriately tailored and culturally sensitive care and facilitate wider use of patient-reported measures. Moreover, failure to incorporate such diversity in patient-reported measures research may exclude and even harm individuals in underserved groups, including those who are racialized, have low literacy levels, are from low- and middle-income countries (LMICs), etc.^13^

The extent to which factors affecting equity of patient experiences and outcomes (e.g., ethnicity, geographic context, socioeconomic status, etc.) and the involvement of service users have been considered in creating and evaluating PROMs and PREMs in psychosis has not been examined. Reviews are used by guideline developers, services, measurement-based systems of care, and researchers to guide their selection of patient-reported measures.^14^ Reviews reflect the state of a field and are often consulted first by individuals desiring to inform and update themselves about a topic^15,16^ and therefore serve as an apt starting point to evaluate the field’s consideration of critical dimensions like equity and inclusion.

Accordingly, our aim was to evaluate existing literature reviews of patient-reported measures on their consideration of sociodemographic factors affecting equity and patient involvement. Specific questions we asked included: To what extent have reviews of PROMs and PREMs in psychosis reported and commented on the sociodemographic characteristics of the populations in their included studies? Have reviews evaluated the readability, costs, and cross-cultural validity of measures? Have reviews commented on whether and how service users were involved in developing patient-reported measures?

## Methods

### Study Design

**Figure 1.**
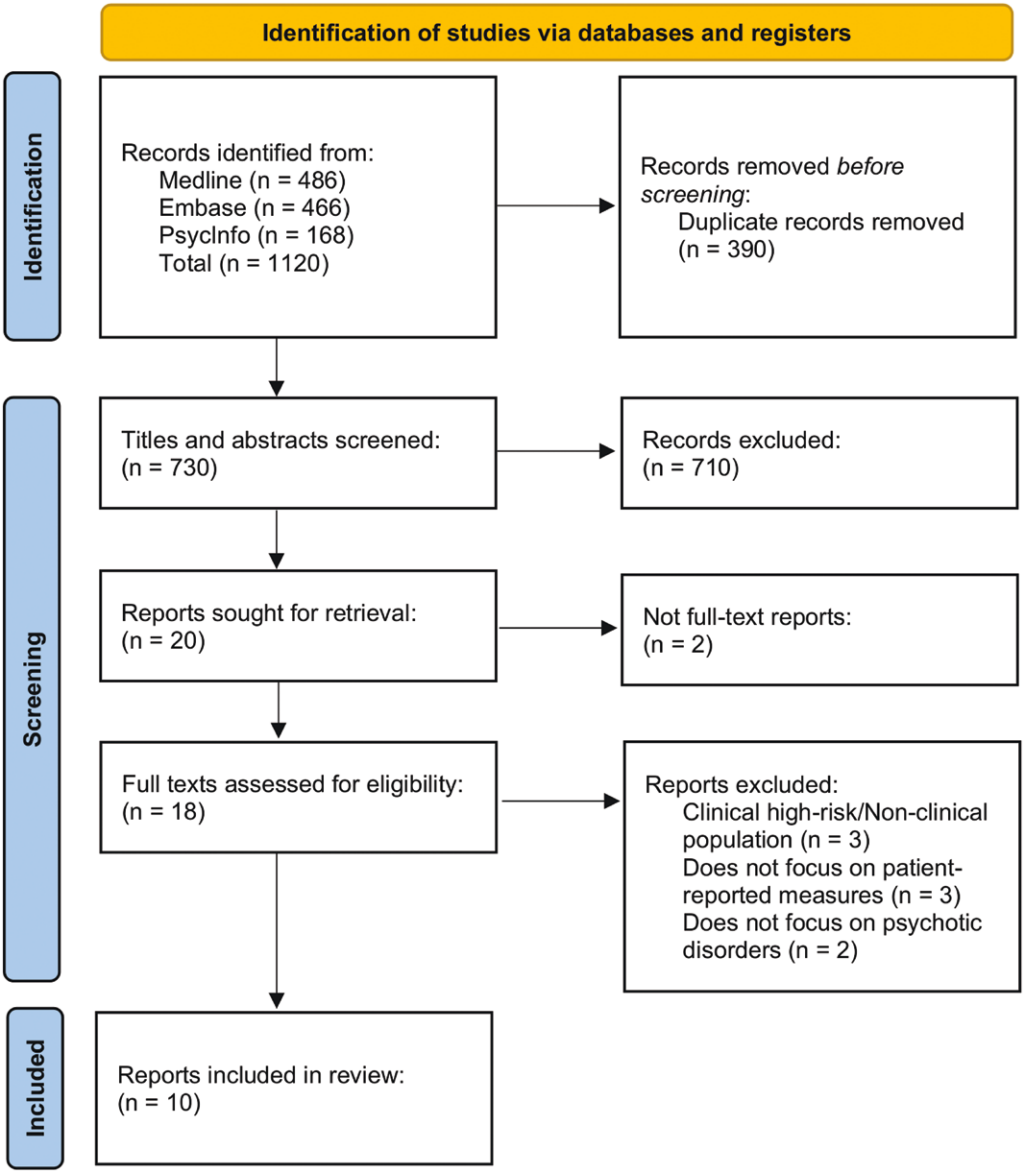
PRISMA Flow Diagram for Identification of Reviews

We conducted a review of reviews (also called overview of reviews)^17,18^ of patient-reported measures in schizophrenia and psychosis and reported it as per PRISMA charting guidelines^19^ (Figure 1).

### Data Sources and Search Strategy

An experienced health sciences librarian (J.B.) developed the search strategy for published reviews on patient-reported outcome and experience measures (and related terms) in schizophrenia and psychosis and performed the literature searches in MEDLINE (Ovid), PsycINFO (Ovid), and EMBASE (Ovid) on May 1, 2023. No date or language limit was applied. The MEDLINE strategy was developed with input from the project team and adapted for use in the other databases. Gray literature was not searched, as the review focused on peer-reviewed publications. The complete search strategy is available at https://doi.org/10.5683/SP3/GCP7IG.^20^

### Eligibility Criteria

Reviews were included if they: (1) they included either studies that examined a particular illness dimension (eg, delusions) or outcome (eg, recovery) assessed via patient-reported or self-reported questionnaires, or studies that used patient-reported or self-reported measures to assess a range of illness dimensions/outcomes; *and* (2) they included studies that focused on schizophrenia, psychosis, and/or other psychotic disorders.

Reviews were excluded if: (1) they focused on clinical high-risk populations or psychotic-like experiences in the general population; (2) their primary focus was a psychiatric diagnosis other than a psychotic disorder or a broad category comprising multiple diagnoses (eg, mental health problems); *or* (3) they were only found as abstracts or conference submissions, rather than as full-text articles.

### Equity and Inclusion Criteria

The criteria for evaluating included reviews were informed by both Cochrane’s PROGRESS-Plus^21^ and the Social Sciences and Humanities Research Council of Canada’s (SSHRC) sections on “Equity, diversity and inclusion (EDI) in research practice” and “EDI in research design.”^22^ As neither framework was judged to be sufficient to answer the study questions, we used a combination of the two to ensure that a more comprehensive equity lens was applied to our review of reviews.

Cochrane’s PROGRESS-Plus is an acronym that stands for Place of residence, Race/ethnicity/culture/language, Occupation, Gender/sex, Religion, Education, Socioeconomic status, and Social capital, while the Plus stands for other characteristics associated with discrimination and/or disadvantage.^21^ This framework was created to identify socially stratifying factors associated with inequalities in healthcare outcomes and services. Using PROGRESS-Plus, we extracted information on whether included reviews considered: Population Diversity, Clinical Characteristics and Health System Context, Accessibility, and Cross-cultural Validity.

The SSHRC equity guidelines focus on the research team’s composition and why, how (approach to analysis/frameworks), and with whom research is done. They align with the equity standards/orientation of other frameworks like the Outcome Measures in Rheumatoid Arthritis Clinical Trials-equity extension^23^ and other agencies including the National Institute of Mental Health,^24^ the National Institute for Health and Care Research,^25^ and the World Health Organization.^26^ Informed by SSHRC guidelines, we extracted information on reviews: Use of Equity Frameworks, Service User Involvement, and Research Team Composition.

### Definitions of Evaluation Criteria

The selected reviews were subjected to an in-depth examination of whether and how they addressed the seven criteria derived from PROGRESS-Plus and SSHRC in their methodology, results (text, tables, and figures), and/or discussion sections.

#### Population Diversity

Informed by PROGRESS-Plus, we examined whether the reviews commented on the place of residence or geographic settings of their included studies, including cities, countries, world regions, and income levels. The reviews were also screened for whether they reported or commented on the age, gender, sexual orientation, race, ethnicity, culture, language, religion, occupation, education, socioeconomic status, and social capital of the included studies’ participants. We also examined whether the reviews identified, within the studies they searched, any other characteristics associated with discrimination/disadvantage or intersecting identities associated with disadvantage (eg, populations who are immigrant and homeless).

#### Clinical Characteristics and Health System Context

We examined whether the review specified the clinical populations of their included studies—specifically, whether they commented on the diagnoses or other characteristics of the illness/illness experience, the clinical setting of the studied population (ie, early intervention, inpatient, outpatient, emergency, residential, etc.) and the health system context (ie, public, private, NGO, etc.).

#### Accessibility

We considered whether the reviews extracted information about the length (number of items, time required) of the PROMs, the ease of administration as determined either by patients or the person administering, the training required to administer the measure, the reading level required by the measure, the literacy of the population that completed the measures, the languages in which the measure was translated and whether the measure had a cost associated with its use.

#### Cross-cultural Validity

We considered whether the reviews examined their articles for psychometric validation for samples from various cultures and/or countries and/or languages.^27^

#### Use of Equity Frameworks

We examined the reviews to see if they used or referred to any frameworks with criteria pertinent to diversity (eg, Consensus-based Standards for the Selection of Health Measurement Instruments [COSMIN]^27^ or any equity-aligned frameworks such as gender-based analysis plus [GBA Plus]^28^), intersectionality and anti-racist approaches to guide their methodology of selecting, reviewing, and/or examining included studies.

#### Service User Involvement

We extracted information on whether and how reviews described and evaluated the involvement of service users (eg, in identifying outcomes; in developing, choosing, or evaluating measures; in designing, implementing, or writing the study, etc.) in included studies.

#### Research Team Composition

There has been growing critique about the limited to nonexistent participation of authors from LMICs in health research, particularly in reviews.^29–33^ Compelling arguments have also been made about the value of lived experience scholarship in mental health and psychosis research.^34^ In both cases, it has been argued that the lack of such inclusion bespeaks epistemic injustice.^34,35^

We therefore extracted information on the income-level and geographic setting of the countries the authors reported being affiliated with. We also looked at whether research teams made explicit the inclusion of any persons with lived experience of mental illnesses and/or psychosis as consultants/co-authors, which was particularly pertinent given our focus on patient-reported measures. See Supplementary Material for our research team’s composition.

### Study Selection Process

All citations were imported into the EndNote X9 software where duplicates were removed. A screening form was developed a priori for study selection and data extraction. We followed a 3-step study selection process. First, 2 reviewers (N.N. and M.A.F.) independently screened titles and abstracts with the screening form. Second, full texts meeting the inclusion criteria were independently reviewed and relevant studies were selected. Third, the same 2 independent reviewers (N.N. and M.A.F.) screened and extracted data from the full texts and a third reviewer (N.K.) resolved discrepancies between reviewers. N.N. and M.A.F. received guidance throughout from N.K. and S.N.I.

### Data Charting and Synthesis

From each review, the following information was extracted: the type of review conducted, the aims of the review, the country in which the review was conducted, the number of measures and studies considered, the psychometric information extracted, and the language restrictions for the inclusion of studies in the review. For studies that did not report the number of studies and measures considered, we calculated this by hand.

The main outcome of interest was whether and how the reviews considered equity and service user involvement (as operationalized by the seven criteria above) when examining their selected studies and forming conclusions such as which patient-reported measures should be used in psychosis and schizophrenia research and care and why.

Data were synthesized descriptively, and study characteristics were presented in tabular form (Table 1). Review characteristics and findings corresponding to each of the examined criteria were formulated into structured summaries by reviewers (N.N. and M.A.F.) with guidance from senior authors.

**Table 1.** Overview of Selected Reviews

Paper	Aim of review	Date of search	Search time frame	Type of review	Country of corresp. author	Psychometric information extracted	Other characteristics evaluated	No. of measures reviewed	No. of studies in review	Language restrictions in inclusion criteria	Language of studies in review
Buck et al. (2022)	To identify psychometric studies of patient-reported measures assessing ongoing symptoms	September 28, 2020	No time restriction	Systematic search and critical review	United States	Internal consistency, test-retest reliability, convergent validity, criterion validity, group differences between clinical and nonclinical individuals, responsiveness to change	Length, symptom domains, presence of assessment of suicidal ideation, scoring methodology, timeframe assessed	11	21	Limited to English-language studies	English
Cavelti et al. (2012)	To review self-report measures for assessing personal recovery	October 2009 to August 2010	No time restriction	Not specified; search strategy defined	Switzerland	Content validity, construct validity, internal consistency, test-retest reliability	User-friendliness, administrator friendliness, translations	13	24^a^	Limited to English-language studies	English
Law et al. (2012)	To examine self-report measures of recovery	Not specified	1990 to present	Systematic review	United Kingdom	Internal consistency, convergent validity, content validity, test-retest reliability	Ease of administration (number of items, time to complete measure, ease of scoring); level of service user input in measure development; service user evaluation	6	12^a^	Limited to English-language studies	English
Krzyzanowski et al. (2022)	To quantify overall and domain-specific self-reported anhedonia	May 22, 2021	Before June 2021	Systematic review and meta-analysis	Canada	Only studies with psychometrically validated measures (metrics unknown) were included	Age, proportion of males, years of education, depression, illness duration, prescribed total antipsychotics, positive symptoms, negative symptoms	15	146	Limited to English-language studies	English
Martins et al. (2016)	To review self-report measures for assessing delusional activity	April 2016	No time restriction	Narrative review with systematic search	Portugal	Internal consistency, test-retest correlation, convergent validity, divergent validity	Issues regarding administration, instructions, number of items, response scale	4	14^a^	Limited to English-language studies	English
McCabe et al. (2007)	To present an overview of patient-reported outcomes	Not specified	Not specified	Selective (nonsystematic) review; search strategy not specified	United Kingdom	Reliability (test-retest, inter-rater consistency, internal consistency); validity (face, predictive, construct, convergent, criterion, discriminant)	Underlying constructs, corresponding scales, and key empirical findings relating to these constructs	19	20^a^	Not specified	Not specified
McKenzie et al. (2022)	To develop a standard minimum set of patient-reported outcome measures that can be used across countries	January to March 2019	January 2013 to January 2019	Systematic literature review	Canada	Reliability (test-retest and internal consistency); validity (content validity, face validity, and construct validity); and responsiveness (sensitivity to change)	Feasibility of collecting these measures, including length of time to administer, training needed, international applicability, wording of items, and previous use in specific populations	131	596	Not specified; only English-language measures included	Not specified
Papaioannou et al. (2011)	To investigate psychometric properties of self-reported quality-of-life measures	August 2009	No time restriction	Systematic review	United Kingdom	Type and method of validity assessment; type and method of responsiveness assessment; validity and responsiveness data	Country of publication, type of disorder, study sample characteristic s (numbers, age, gender), other measures used	4	33	Limited to English-language studies	English
Millier et al. (2014)	To identify all patient-reported outcome questionnaires used in evaluating patients with schizophrenia	Not specified	Not specified	Systematic review	France	Internal consistency, reproducibility, content validity, construct validity, sensitivity to change	Target population (generic or specific), dimensions, languages in which developed or translated, the number of items, whether the article was dedicated to validation or only mentioned validity	73	70	No language restrictions (translations were done when required)	Spanish, French, English, Japanese, Chinese, Finnish, Hebrew, Arabic, Danish, German, Greek, Irish, Italian, Dutch, Portuguese, Swedish, Turkish, and Korean
Reininghaus et al. (2012)	To examine measures of 4 widely used patient-reported outcomes in the evaluation of care of people with psychosis	Not specified	Not specified	Conceptual and methodological review; systematic search	United Kingdom	Internal consistency; reliability; scale information; content validity; construct validity (structural, convergent, discriminant, cross-cultural, concurrent, predictive validity); responsiveness	Concept to be measured, number and content of domains, estimated completion time, response options, type	37^a^	175	Not specified	Not specified

^a^The number of studies and/or measures were hand-counted for reviews that did not report them.

### Quality Assessment

A quality appraisal was conducted to assess the overall quality of the included reviews. Given our focus on equity, we wanted to be inclusive and did not eliminate any reviews based on their quality. The included systematic reviews were appraised for their quality using the 11-question JBI Critical Appraisal Checklist for Systematic Reviews and Research Syntheses.^36^ This appraisal assessed the search strategy, the efforts adopted to minimize bias, and whether the reviews included recommendations for practice and future directions. Each question was answered with a “yes,” “no,” “unclear,” or “NA.” Nonsystematic reviews were appraised using the 6-question Scale for the Assessment of Narrative Review Articles (SANRA).^37^ This tool assessed papers for their logical structuring and methodological strategy and each question was rated on a scale of 0-2. Before conducting the appraisals, the reviewers (N.N. and M.A.F.) discussed and established what each evaluation criterion comprised. The appraisals were conducted independently by each reviewer and a consensus was reached with the help of a third reviewer (N.K.). Positive answers (“yes” for systematic reviews and a score of 2 for nonsystematic reviews) were tallied, the results of which were tabularized (see Tables 2 and 3).

**Table 2.** Quality Appraisal for Systematic Reviews

	Buck et al. (2022)	Law et al. (2012)	McKenzie et al. (2022)	Millier et al. (2014)	Papaioannou et al. (2011)	Krzyzanowski et al. (2022)
1. Is the review question clearly and explicitly stated?	Yes	Yes	Yes	Yes	Yes	Yes
2. Were the inclusion criteria appropriate for the review question?	Yes	Unclear	Unclear	Unclear	Yes	Yes
3. Was the search strategy appropriate?^a^	Unclear	Unclear	Unclear	Yes	Unclear	Unclear
4. Were the sources and resources used to search for studies adequate?	Yes	Yes	Yes	Yes	Yes	Yes
5. Were the criteria for appraising studies appropriate?	No	No	No	No	Yes	No
6. Was critical appraisal conducted by 2 or more reviewers independently?	No	No	No	No	No	No
7. Were there methods to minimize errors in data extraction?	Yes	No	No	Yes	Unclear	Yes
8. Were the methods used to combine studies appropriate?	Yes	Yes	Yes	Yes	Yes	Yes
9. Was the likelihood of publication bias assessed?	N/A	N/A	N/A	N/A	N/A	Yes
10. Were recommendations for policy and/or practice supported by the reported data?	Yes	Unclear	Yes	No	No	No
11. Were the specific directives for new research appropriate?	Yes	Yes	Yes	Yes	Yes	Yes

^a^This does not mean that the search strategy was not appropriate, but that the authors may not have adequately justified choices like restricting their search to English or the timespans on which their search focused.

**Table 3.** Quality Appraisal for Nonsystematic Reviews

	McCabe et al. (2007)	Martins et al. (2016)	Cavelti et al. (2012)	Reininghaus et al. (2012)
1. Justification of the article’s importance for the readership	1	2	2	1
2. Statement of concrete aims or formulation of questions	2	2	2	2
3. Description of the literature search	0	2	2	2
4. Referencing	2	2	2	2
5. Scientific reasoning	2	2	2	2
6. Appropriate presentation of data	2	2	2	2

## Results

### Search Findings

Our search yielded 1120 results, and after removing duplicates and screening titles, abstracts, and full texts, 10 reviews were finally included in our review of reviews (Figure 1).

#### Types of Reviews

The 10 reviews, including 6 systematic and 4 nonsystematic reviews, represent a total of 1111 studies (range of 12-596 studies) and 313 measures (range of 4-131 measures). See Table 1 for a summary of the included reviews. Six reviews reported the number of studies reviewed but 4 did not.^38–41^ One review^42^ did not report the number of measures considered. These were included in our totals after calculating these from the papers.

All the reviews had a strong focus on the psychometric properties of the measures they considered. Six reviews^38–40,43–45^ focused on patient-reported measures of specific domains. Specifically, 2 reviews examined self-report measures of recovery in psychosis and focused on their measurement qualities, with the goal of helping services identify the best-suited patient-reported recovery measures.^38,39^ One review focused on studies of the psychometric properties of patient-reported measures of schizophrenia symptoms,^43^ while another looked at patient-reported measures of the features and gradations of delusional ideation.^40^ Another review included studies with psychometrically validated self-report measures of anhedonia in persons with schizophrenia.^44^ One review solely focused on 4 quality-of-life patient-reported measures and documented their psychometric evidence across studies.^45^

The remaining 4 reviews were broader in scope, focusing generally on patient-reported measures in psychosis, including but going beyond only their psychometric properties.^41,42,46,47^ One of these, McKenzie et al.,^47^ developed a set of PROMs for psychotic disorders, using a modified Delphi process and a systematic literature search. The review was 1 component of this study and therefore only briefly detailed.

### Main Findings

#### Population Diversity

Three of the 10 reviews^42,46,47^ neither extracted nor commented on the size or characteristics of the populations in the studies they reviewed. The remaining 7 reviews commented on population characteristics at varying levels of detail.^38–41, 43, 45, 47^ Three^38,40,43^ only reported the sample size of the final studies reviewed, without mentioning any other demographic data.

Two reviews, in addition to reporting on sample size, also reported the country the samples came from for validation studies.^39,45^ In both these reviews, the validation studies were mainly conducted in high-income countries, apart from 2 studies that took place in Ethiopia.^45^ However, neither of the 2 reviews commented on this lack of geographical and socioeconomic diversity.

Only 2 reviews reported on the age and gender distributions of the samples considered; however, no insights were drawn about the proportions of various genders in the sample or how many studies viewed gender solely as a binary.^44,45^ One review commented on whether age, gender, ethnicity, race, employment, and education level influenced levels of empowerment and self-esteem, as measured by 2 specific self-report scales. This review did not comment on these characteristics in relation to any of the other examined self-report measures.^41^

None of the reviews systematically extracted or commented on the language of the target populations of their selected studies, nor did they comment on the race/ethnicity/culture, occupation, religion, socioeconomic status or social capital of the populations or the income level or world region in which their selected studies were conducted. Only 1 review extracted information about the years of education of the participants in their included studies and used this to predict social anhedonia; however, no comments were made to otherwise characterize study populations in their included studies.^44^

Moreover, none of the reviews extracted information or commented on vulnerable populations or populations experiencing discrimination or intersecting disadvantages in their included studies, except for one listing of an individual study focusing on “individuals experiencing or at risk for homelessness.”^43^

While McKenzie et al.^47^ did not extract information about the population diversity in their included studies, they provided a list of demographic and clinical “risk-adjustment factors” that could affect patient-reported outcomes, which includes most of the PROGRESS-Plus characteristics (except for a place of residence, religion, and social capital) and additional ones (eg, sexual orientation, hospitalizations, comorbidities, adverse life experiences).

#### Clinical Characteristics and Health System Context

While all reviews specified schizophrenia, psychosis, or schizophrenia-related disorders in their search/inclusion criteria, only 3 reviews systematically reported, for each included study, the specific diagnoses (ie, affective/non-affective, schizophreniform, etc.) of the studied populations and their clinical setting.^40,43,45^ One review only partially reported on both the clinical diagnoses and the setting where the populations were recruited from in the validation studies it examined and occasionally left out these details completely.^38^ Two reviews reported on neither of these for their included studies.^42,47^

One review reported on the diagnoses of the clinical populations in each included study,^39^ while another review extracted information about illness duration but did not comment on this further.^44^ The remaining 2 reviews mentioned the target population for which the measures had been designed (ie, whether they were generic, schizophrenia-specific, mental illness-specific, etc.); however, they did not specify the diagnoses or clinical settings of the populations examined within the selected studies.^41,46^ None of the reviews extracted information about or commented on the health system context or any other descriptors of the illness/illness experience of the patient populations in their included studies.

#### Accessibility

Nine out of 10 reviews commented on the number of items included in the patient-reported measures they reported on. Five reviews considered the length of time required to complete a given self-report measure.^38,39,41,42,47^ Four reviews extracted information on the response options/formats used by the measures,^38,40,42,43^ while 2 reviews investigated issues regarding formatting (e.g., variations in answer formats for the same patient-reported measure).^38,40^

Five reviews reported on the ease and accessibility of administering and scoring measures.^38,40,43,47^ However, only 2 studies within these 5 reviews contacted actual service users to assess how simple the format was and how easy the scoring was to understand^39^ or to provide feedback with regard to the wording and appropriateness of measures.^47^ McKenzie et al.^47^ considered training required to administer measures. None of the reviews commented on the method used by included studies to assess the ease/accessibility of responding to measures. The reviews themselves also assessed “ease” with their own predetermined criteria. Two reviews examined the language of the patient-reported measures, specifically whether the questions were negatively formulated (as this was shown to lead to poorer comprehension) or if item or answer formats within a measure varied from item to item^38^ and whether the language was deemed “positive and acceptable” by the 2 service users on the research team.^39^

None of the reviews commented on the literacy levels of the populations that the patient-reported measures were used with, and only 1 briefly mentioned that one of the patient-reported measures they focused on—the Conviction of Delusional Beliefs Scale—was written at a fifth-grade reading level.^41^

As for translations, 4 reviews extracted the languages into which the patient-reported measures had been either developed or translated.^38,40,44,46^ Only 1 review did not restrict its search by language and translated articles when necessary and hence, reviewed various patient-reported measures in 20 languages.^46^ They also found that the most translated PROM was the Schizophrenia Quality of Life Scale Revision 4 (SQLS-R4), which had been translated into 52 languages through standardized procedures, although neither the psychometric properties of the translated versions, nor the quality or nature of translation procedures used, were commented on.^46^ Only 2 reviews systematically examined whether the psychometric reliability and validity of the patient-reported measures had been established in the translated versions,^38,40^ while only one considered whether those translated versions had been psychometrically validated.^44^ Out of the thirteen self-reported recovery scales assessed, Cavelti et al.^38^ found that only 3 had been translated (into one other language each) and that the psychometric properties of the translated versions had been evaluated. Of the 10 remaining patient-reported measures in this review, 7 had no translated versions and 3 had translated versions whose psychometric properties had not been assessed. The Martins et al.^40^ review examined the psychometrics of translated versions (Korean, French, Spanish, German, Italian, and Taiwanese) of one of their patient-reported measures, the Peters Delusions Inventory. As for the Krzyzanowski et al. (2021)^44^ review, only 7 out of the 15 measures examined had been translated in included studies, and across these, translations were limited to English, Chinese, French, and German.

Only 2 reviews^38,47^ considered the cost of using measures. Cavelti et al.^38^ extracted data about the accessibility of measures and Supporting Materials (ie, copyright, user fees, free availability online or upon contacting authors). McKenzie et al. only considered measures available without a cost for their final set. Only 1 review^38^ considered modes of administration (paper-pencil, computer, or internet) and data processing of included patient-reported measures, which are important considerations in implementing measures.

#### Cross-cultural Validity

Only 1 review extracted data on cross-cultural validity. It reported that only 2 included studies (focused on the EuroQOL-5D and the Client Assessment of Treatment scales) out of a total of 175 included studies assessed this property.^42^ Another review briefly mentioned that 6 of its included studies had been undertaken across more than 1 country but did not comment on this further in terms of validity, adaptations of measures, etc.^45^ Two reviews commented on how using measures that have been translated and linguistically and psychometrically validated, using standardized methods, could help compare data from patient-reported measures across distinct cultures and languages.^38,46^ Moreover, 2 reviews commented on cultural variations in the conceptualization of certain themes (ie, recovery), but neither extracted information from their studies of recovery measures on cross-cultural variability in conceptualizations or validity.^38,39^ McKenzie et al.^47^ considered the “international applicability” of measures but did not elaborate on how this was operationalized.

#### Use of Equity Frameworks

While 2 reviews used the COSMIN framework,^42,47^ none of the reviews made any reference to any equity-aligned frameworks or approaches to examining the studies in their reviews. Also, no reviews commented on or recommended the use of such frameworks in research on patient-reported measures.

#### Service User Involvement

Only 1 review extracted information regarding the “level of service user input during [the] development of the measure,” considering factors such as the inclusion of service user researchers and service user feedback in the development of the measure.^39^ This review identified 6 measures of recovery and synthesized, in a tabular form, the ways in which each one incorporated service user input in its design. All 6 measures examined in this review included service user input; however, the input took various forms for each measure, including focus groups, feedback on content and wording, service users in the research team, pilot testing by service users, or some combination of the aforementioned. None of the other reviews commented on service user involvement in the included studies, even when they considered factors such as user-friendliness or accessibility.

Although McKenzie et al.^47^ did not describe or evaluate the involvement of service users in the patient-reported measures they found and considered for their international set of recommended measures, their own process involved service users (and caregivers) in identifying outcomes that were important to them through multiple methods (focus groups, Delphi, breakout sessions).

#### Research Team Composition

Authors of all but 2 of the reviews were affiliated only with institutions in high-income countries: Australia, Canada, Denmark, France, Germany, Israel, Netherlands, Portugal, Switzerland, the United Kingdom, and the United States. Millier et al.^46^ included 2 authors (out of 7 authors) associated with an institution in Tunisia. McKenzie et al. had a working group with members from 11 countries, including 2 LMICs (India and Mexico). These same 2 countries were also the only LMICs represented in their authors’ list (2 out of 26 authors). Notably, McKenzie et al.^47^ also listed 2 service users as co-authors (one from an LMIC).

Only 1 other review^39^ included 2 service users/persons with lived experience in their research team and as co-authors. These 2 members identified criteria that were important for them when completing a self-report, completed all measures themselves, and provided feedback along the identified criteria that were presented in the review. No information about the service user co-authors’ (or other authors’) backgrounds, specific experiences, or other aspects were noted. None of the reviews make any reference to equity or inclusion considerations within their own research team.

### Quality Appraisal

The 6 systematic reviews fulfilled between 4 and 7 of the 11 criteria on the quality appraisal checklist, with many answers marked as “unclear” (Table 2). Out of these, 5 did not perform quality appraisals, while one carried out a quality appraisal that neither used a formal quality assessment checklist nor used 2 or more reviewers.^45^ Law et al.^39^ used criteria identified by 2 service user-authors to evaluate included measures but did not frame this as “quality appraisal” or use items in typical quality appraisal tools.

None of the reviews that only considered English-language studies provided justifications for this inclusion criterion or listed it as a limitation. Three of the reviews used appropriate methods to minimize bias,^43,44,46^ while one was unclear if it did^45^ and another 2 reviews did not mention any such methods.^39,47^ While all 6 systematic reviews provided suggestions for future research, only 2 provided clear recommendations for policymakers and practitioners.^43,47^ No clear associations were found between those systematic reviews that scored better on the JBI criteria and those that considered more equity and inclusion criteria.

The 4 nonsystematic reviews scored between 9 and 12 (out of a possible 12) on the quality appraisal checklist for nonsystematic reviews, with 2 receiving full scores^38,40^ (Table 3). All 4 nonsystematic reviews provided well-formulated aims, consistently backed up their claims with references, and appropriately presented their data. Two did not explicitly justify their importance for readership,^42,43^ while one did not present any search strategy.^41^ Those nonsystematic reviews that scored all 12 points did a better job at considering targeted equity criteria compared with those that scored lower on the SANRA.

## Discussion

We found that reviews of patient-reported measures in psychosis demonstrate a lack of attention to equity and the involvement of service users. Although patient-reported measures are increasingly used and advocated for, they cannot be assumed to reflect a truly patient-centered approach unless they assess perspectives that patients have prioritized during their creation. Our findings also underline the concern that many measures, while patient-reported, may not be accessible, acceptable, valid, or even culturally safe for patients from distinct demographics, communities, and countries. At the very least, that reviews rarely commented on the sociodemographic characteristics of populations in included studies means that critical knowledge gaps remain about the availability and suitability of patient-reported measures for demographically, linguistically, geographically, and socioeconomically diverse populations.

### Service User Involvement as Primordial but Neglected

Only 1 review^39^ extracted information about how service users were involved in developing patient-reported measures. This review focused on personal recovery measures. Pointedly, no reviews of self-reported symptom measures included any consideration of service user involvement. In thus generally undervaluing service user involvement, the study of patient-reported measures is ironically turning away from what propelled the uptake of such measures in the first place: the epistemological shift toward regarding patient experience and priorities as a fundamental form and source of knowledge.^3^ How patient centered can patient-reported measures (and measurement) be if we cannot even tell whether and how patients were included in their conception, design, and evaluation?

Our finding is consistent with a scoping review of measures used in early intervention for psychosis.^48^ Although this review was not limited to PROMs and PREMs, only one out of its 115 studies included information on service user involvement in the selection or implementation of measures.

### Minimal Attention to Equity-Influencing Factors

Despite increasing expectations to incorporate equity into psychiatric research practices and policy,^49–51^ most reviews (including some of the later ones) neither adequately characterized the samples represented in their included studies (e.g., countries, gender) nor commented on how a lack of diversity in samples and the predominance of Western, Educated, Industrialized, Rich and Democratic (WEIRD) countries could be a limitation of their work or the included studies.

All reviews extracted psychometric information from the measures they evaluated, focusing on various kinds of reliability and validity. In doing so, the reviews demonstrated the priority they accorded to psychometric characteristics as a primary determinant of a patient-reported measure’s suitability. Even within these psychometric characteristics, cross-cultural validity—a more diversity-focused psychometric property—was only considered once, revealing that little attention is paid to how measures perform across diverse cultures and contexts. When measures were reported to be validated in different countries, the countries were usually of high-income status, matching previous findings that high-income countries are overrepresented in schizophrenia research.^51^

Dimensions that are critical for equitably scaling up measurement-based care and open science were rarely considered, such as the cost, availability in different languages, and literacy or readability demands of measures.

Although developing a set of PROMs for psychosis for international use, McKenzie et al.^47^ restricted themselves to measures available in English and did not comment on the availability in different languages of the measures in their final set. The few reviews that commented on the different languages in which patient-reported measures were available did not report on whether best practices for translating measures were followed, even though various guidelines are available to both translate and culturally adapt patient-reported measures.^52,53^

All patient-reported measures in the included reviews were developed for literate populations. Across health fields, it has been recommended that the readability of patient-reported measures be assessed and considered in their selection.^11,54,55^ Doing so may be even more important for persons with psychosis, many of whom have educational attainment gaps and neurocognitive difficulties.^56–58^

In terms of ease of use, the reviews mostly focused on quantitative criteria such as the number of items in a measure and the length of time required to complete it. While these characteristics are important, they are not enough to judge the acceptability of measures for patients.

Gender, urbanicity, immigration status, economic level, and other social and environmental factors have been shown to significantly affect the incidence and course of psychosis.^59,60^ The psychosis diagnoses of ethno-culturally diverse individuals have been found to have been mistaken upon reassessment with cultural formulations.^61^ Taking this into consideration, we need patient-reported measures that are suitable for a wide range of sociodemographics. It is also essential that reviews of patient-reported measures consider this criterion when commenting on the state of the field. When not vetted by or validated for specific populations, patient-reported measures that guide treatment decisions can end up producing harmful repercussions.

### Representation in Research Practice

All 10 examined reviews were written and published in English. Eight were by authors in high-income, Western countries, highlighting the limited representation of LMICs within the research teams reviewing patient-reported measures in psychosis. The only one^39^ of the 10 reviews that commented on whether service users were involved in developing patient-reported measures was also one of the only 2 reviews^39,47^ that included service users in their teams. It cannot be definitively concluded that representation in research teams of LMICs and service users would automatically translate into research that is more representative of diverse contexts and more patient centered. Nonetheless, the need for representation in research practice extends far beyond its instrumental value.^62,63^

### Strengths and Limitations

Our review is novel in its approach of using a comprehensive, holistic range of criteria to evaluate the ways in which existing reviews of patient-reported measures have prioritized equity and inclusion. Our study also benefits from the inclusion of a systematic quality appraisal of the included reviews. However, it should be noted that neither the JBI nor the SANRA checklists themselves explicitly consider and include equity and inclusion criteria. Also, the positionality of authors (and whether this is stated) is not even part of either checklist. All steps were carried out by at least 2 reviewers, which helped reduce potential bias in the search, selection, and extraction processes.

In terms of limitations, we included both systematic and nonsystematic reviews of varying quality and breadth of scope. While this precluded our ability to compare reviews, being thus inclusive allowed us to comment more generally on the field. We did not notice any clear pattern between the quality/scope of reviews and their consideration of equity and patient involvement. A key shortcoming of only reviewing reviews themselves is our limited ability to infer if the lack of consideration to equity and lived experience we noted also extends to the design of each of the individual studies within the selected reviews. Still, we can safely critique the state of literature reviews on patient-reported measures, acknowledging that reviews often shape practice/guidelines. A valuable next step would be a systematic, high-quality review of patient-reported measures in psychosis informed by PROGRESS-Plus and other equity criteria. Although we did not restrict our search by language, we used 3 databases in which English-language studies predominate. Our review may have thus not picked up reviews in other languages. Our search was also limited to reviews published in larger peer-reviewed databases. Future reviews should consider accessing gray literature and a wider variety of databases.

### Implications

To help clinical and research programs in psychosis identify the patient-reported measures that are best suited for their target populations, reviews must report information on factors affecting equity and comment on associated gaps. Based on the criteria we used to evaluate reviews and the gaps we identified, we generated a list of recommendations for future reviews of patient-reported measures so they can be more equity- and inclusion-focused (Table 4). More structured methods are needed to formalize these into guidelines.

**Table 4.** Equity- and Inclusion-Focused Recommendations for Future Reviews on Patient-Reported Outcome Measures

Population diversity	Extract and report sociodemographic information about the patients included in the studies (including age, gender, sexual orientation, race, ethnicity, culture, language, religion, occupation, education, socioeconomic status, social capital, etc.). Extract and report information on the geographic and socioeconomic context in which the study was conducted (including city, country, world region, income level (high or low-middle), etc.). Comment about gaps in population diversity in included studies.
Clinical characteristics and health system context	Report on the clinical setting (inpatient, outpatient, emergency, residential, etc.) and health system context (public, private, nonprofit) from which the patients in each study were included. Specify the diagnoses, comorbid conditions, adverse life experiences, and service utilization history (eg, hospitalizations) of the patients included in the studies. Specify the interventions that patients included in the studies received. Comment about gaps in the study population (clinical characteristics) and clinical setting/health system context.
Measures of accessibility	Extract and report information regarding the readability levels of PROMs, referring to tools used to assess and report readability such as the Flesch-Kincaid reading grade^64,65^ or the Simple Measure of Gobbledygook.^66^ Extract and report information on the known literacy rates and reading levels of the population in which the tool was validated. Systematically report the languages that PROMs have been translated into and validated in. Systematically report on the costs involving in using measures and prioritize measures available easily and free of cost. Extract information on the training required to administer measures and whether measures are available and have been tested in paper-and-pencil and computer versions. Comment on gaps related to measures of accessibility.
Cross-cultural performance	Evaluate studies for whether they report on cross-cultural reliability and validity of their PROMs and PREMs, either across different linguistic translations or across various cultural backgrounds. Evaluate any other ways in which studies assess applicability across contexts/samples.
Use of existing EDI frameworks	Refer to equity/social determinants' frameworks and incorporate equity-oriented tools, such as the PROGRESS-Plus criteria^22^ and the 8Quity tool^67^ or the consensus guidelines on minimum standards for patient-reported outcome measures of the International Society for Quality of Life Research.^53^ Comment on whether and how included studies/measures refer to equity frameworks or frameworks or tools aligned with diversity, including the EMPRO.^61^
Service user involvement	Extract and report on whether and how studies of patient-reported measures include service users (and for studies that include service users, the level of details they provide as to type and level of involvement). Consider service user involvement as an essential criterion for patient-reported measures and, accordingly, appraise included studies and comment on their limitations in relation to service user involvement.
Research team composition	Thoughtfully include persons with lived experiences of mental ill health/mental health services in the research team and processes.^35,68^ Meaningfully integrate multiple perspectives and reflexivity statements from authors from diverse backgrounds (ie, from WEIRD and non-WEIRD countries, with intersecting identities, different career stages, etc.).^35,69^
Review methodology	Do not limit searches to English or at least, provide clear justification for restricting review to English or any other language. To the extent possible, include articles published in as many languages as possible to capture PROMs developed and tested in diverse contexts and languages. Use appropriate quality appraisal tools.

Abbreviations: PREMs, patient-reported experience measures; PROMs, patient-reported outcome measures; WEIRD: Western, Educated, Industrialized, Rich and Democratic.

Peer reviewers and journal editors must ensure that studies and reviews of patient-reported measures systematically include and report setting and population characteristics such as those in PROGRESS-Plus and the risk-adjustment factors listed by McKenzie et al.^21,47^

With respect to service user involvement, at a minimum, measure development and validation studies and reviews of measures should describe the presence/absence of service user involvement, and where present, provide sufficient details as to the type and level of involvement. Patient-reported outcome and experience measures developed with no identifiable service user involvement should be viewed as only partially aligned with the actual goals of patient-centered care and patient-centered outcomes research.^10^

Some international groups, such as the International Society for Quality of Life Research, have issued consensus guidelines on minimum standards for patient-reported measures, including attending to the diversity of participant samples, and evidence that “patients…consider the content of the PRO [patient-reported outcome] measure relevant and comprehensive.”^53^ Similarly, EMPRO, a standardized tool developed by a Spanish research group for assessing PROMs, explicitly includes and addresses cross-cultural and linguistic adaptations.^70^ Despite these efforts, psychiatry and mental health journals appear to be publishing measurement and measure review articles with minimal attention to cultural and geographic diversity and the explicit inclusion of service users.

A potential driver of inattention to equity and diversity is the sustained biomedicalization of schizophrenia,^71,72^ that is conducive to assuming cross-cultural similarities in core psychopathology^73,74^ and neglecting the social and structural determinants of psychosis outcomes.^75,76^ It is encouraging that McKenzie et al.^47^ recommended that clinicians and researchers assess several demographic and clinical factors while deploying the patient-reported outcomes and measures that they compiled for international use. However, their characterization as “risk-adjustment factors” reveals a view of sociodemographic and clinical factors as mere predictors or covariates and not as essential to the identities, experiences, and socio-environmental contexts of persons with psychosis.

If the inclusion of patients is not a key barometer by which the conception, design, and performance of patient-reported measures are evaluated, these measures or their use may not have the desired effects. The fuller and wider inclusion of patients may also help redress some of the glaring equity gaps with respect to language, region, income, education level, etc. that still vex psychosis research.^77^ More fundamentally, a rights- and values-based case can and must be made for the deep involvement of service users in designing, choosing, and implementing patient-reported measures, and in reviews and evaluations of such measures.

## Supplementary Material

sgae032_suppl_Supplementary_Material
