# Supplementary material for: A Review of Reviews of Patient-Reported Measures in Psychosis: Need to Consider Factors Affecting Equity and the Involvement of Patients
Source: Schizophr Bull Open. 2025 Jan 11;6(1):sgae032. doi: 10.1093/schizbullopen/sgae032 (PMC11920872; doi:10.1093/schizbullopen/sgae032)
Supplement: sgae032_suppl_Supplementary_Material [file sgae032_suppl_supplementary_material.docx]

**Supplement**

**Research team composition**

We constructed our team to include scholars with content and methodological expertise from high-income and low-and middle-income contexts and varying career stages, and scholars with lived experience of psychosis and of immigration and racialization. Our team includes a White scholar with lived experience of psychosis (NJ); scholars from low- and middle-income countries (GM) and with research and clinical experience in low- and middle-income countries (GM, SNI) ; scholars with lived experience of immigration (NN, M-AF, NK, SNI) and belonging to visible minority groups (NN, NK, SNI); scholars with experience in psychosis services and research (NN, GM, NJ, SNI); and scholars with experience in literature review methodologies (NK, SNI). NN and M-AF are trainees; NJ and NN are early-to-mid career scholars; and SNI is a senior scholar.
